# Lectures on Diseases of the Heart

**Published:** 1888-05

**Authors:** 


LECTURES ON DISEASES OF THE HEART. By Alonzo Clark, M. D., LL. D. E. B. Treat & Co., 771 Broadway, New York, 1887.

Dr. Alonzo Clark has been for many years a lecturer at the College of Physicians and Surgeons, of New York. This book is composed chiefly of lectures delivered at that college. It is emphatically the work of an original thinker and worker—not in any sense a compilation.

